# Effect of Antiseizure Medication on the Salience Network in Patients with Epilepsy with Generalized Tonic-Clonic Seizures Alone

**DOI:** 10.3390/biomedicines12071521

**Published:** 2024-07-09

**Authors:** Cătălina Elena Bistriceanu, Georgiana-Anca Vulpoi, Iulian Stoleriu, Dan Iulian Cuciureanu

**Affiliations:** 1 Neurology Department, Faculty of Medicine, University of Medicine and Pharmacy “Grigore T. Popa”, 16 Universitatii Street, 700115 Iasi, Romania; vulpoi.anca@yahoo.com (G.-A.V.); cuciureanudan@yahoo.com (D.I.C.); 2 Elytis Hospital Hope, 43A Gheorghe Saulescu Street, 700010 Iasi, Romania; 3 Dorna Medical, 700022 Iasi, Romania; 4 Faculty of Mathematics, “Alexandru Ioan Cuza” University, 11 Bd. Carol I, 700506 Iasi, Romania; stoleriu@yahoo.com; 5 Neurology Department I, “Prof. Dr. N. Oblu” Emergency Clinical Hospital, 2 Ateneului Street, 700309 Iasi, Romania

**Keywords:** epilepsy with generalized tonic-clonic seizures alone (EGTCSa), salience network, low resolution electromagnetic tomography (LORETA), antiseizure medication (ASM)

## Abstract

This study aimed to investigate the effects of antiepileptic drugs on salience network regions in patients with epilepsy with generalized tonic-clonic seizures alone (EGTCSa). A retrospective observational case-control study was performed on 40 patients diagnosed with epilepsy with EGTCSa and 40 healthy age-matched controls. In LORETA, a voxel-by-voxel analysis between regions from the salience network was performed for both hemispheres, specifically between the anterior cingulate (BA 32 and BA 24) and the sublobar insula (BA 13). Subsequently, a Wilcoxon rank-sum test (the Mann-Whitney U test) was conducted for the equality of medians in the transformation matrix. A comparison was then made between each region of interest as defined by the salience network and the controls. Marked differences were found in the brain regions assessed in patients with EGTCSa treated with valproic acid and carbamazepine compared to the control group; few differences in patients treated with levetiracetam; and no difference was found in the group without treatment compared with those in the control group. These results suggest that ASMs can influence cognitive processes, which provide novel insights toward understanding the neural mechanisms underlying the effects of ASMs administration.

## 1. Introduction

Idiopathic generalized epilepsies (IGEs) include childhood absence epilepsy, juvenile absence epilepsy, juvenile myoclonic epilepsy, and epilepsy with generalized tonic-clonic seizures alone (EGTCSa) [1]. Around 8–10% of IGEs are represented by juvenile absence epilepsy. It manifests with severe typical absences and usually onsets in children between 9 and 13 years old. Juvenile myoclonic epilepsy is one of the most important syndromes of IGEs. Most patients have the following types of seizures: myoclonic jerks on awakening (all), generalized tonic-clonic seizures (over 90%), and absence seizures (about one-third) [2,3,4].

Among IGEs, the prevalence of idiopathic generalized epilepsy with GTCS varies from 10–15%, as reported in a French department of epilepsy [2], to 62%, as reported in an Epilepsy Program from South Florida [3,4].

Earlier descriptions of EGTCSa were published in 1953 by Janz, who described generalized tonic-clonic seizures (GTCS) occurring principally on waking or during leisure (evening) [5]. Whilst IGE with GTCS on awakening became universally recognized in 1989, the International League Against Epilepsy followed up by including patients with random and nocturnal GTCS later [6]. A minimum of six GTCS are required to diagnose epilepsy with grand mal on awakening or grand mal predominantly during sleep [5].

The salience network includes the bilateral anterior insula, dorsal anterior cingulated cortex, and the anterior temporo-parietal junction. There has been evidence that this network interacts with complement functional networks in the brain for guiding behavior. Specifically, the anterior insula and the anterior cingulate cortex are responsible for guiding behavior, mainly by separating internal and external stimuli [7,8].

In childhood absence epilepsy, previous studies have found altered salience network connectivity (increased connectivity in the anterior and middle cingulate gyrus and caudate nuclei) [7,8].

The salience network functions by switching between self-directed attention and directed attention on the outside world. Mechanistically, it represents a dynamic change between the default mode network and the frontoparietal network [9].

It is believed that the anterior cingulate cortex plays an important role in the cognition, affection, and seizure propagation of patients with epilepsy. Children with epilepsy show a better neurocognitive outcome when resting state networks are not disrupted by interictal epileptiform discharges. In this regard, the normalization of intrinsic network connectivity through brain stimulation procedures might improve cognitive outcomes in epilepsy patients [10].

As a result of anti-seizure medications (ASM), certain brain regions may be affected. Previous studies on neural networks have speculated that ASMs may alleviate the abnormal coupling of functional and structural networks [11]. However, numerous patients with epilepsy were found to exhibit an excessively activated salience network in the anterior cingulate cortex. A failure of the salience network to integrate the relevant internal and external stimuli, coupled with the inability to interconnect with other functional neural networks that regulate behavior, may explain the deficits of consciousness and attention experienced by such patients [12].

In addition, other studies on patients with right temporal lobe epilepsy have previously found the abnormal modulation of the salience network. In such patients, the anterior cingulate cortex and bilateral anterior insula showed disrupted connectivity [13].

The salience network serves an important role in communication, social behavior, self-awareness, and cognition. Salience network alteration (along with the default mode network and fronto-parietal attention network) has been found in patients with chronic temporal lobe epilepsy and idiopathic generalized epilepsy, which is inferred to explain the cognitive impairments reported in these patient groups [14].

A number of connectivity studies using functional MRI (fMRI) have reported that differences in the network features between groups with IGE and healthy controls are due to effects resulting from the medications. In another study, carbamazepine was found to alter ‘betweenness centrality’ (but not other network metrics) compared with that in other commonly used ASMs [15,16].

The electroencephalogram (EEG) is a commonly used non-invasive technique in epilepsy research. Despite its low cost and applicability, the EEG device has several disadvantages, including its performance limitations as a substantial amount of data must be processed and stored. Obtaining artifacts (physiological or environmental) also requires appropriate qualification and correction, sometimes preventing a correct interpretation of results. The EEG remains the primary method for assessing epilepsy and sometimes combining it with functional MRI or additional genetic testing can yield useful information.

A possible approach to classical analysis based on the visual inspection of interictal epileptiform discharges could be to use the resting-state brain activity of patients with generalized epilepsy to complement classical analysis [17].

This study aimed to investigate whether antiepileptic drugs can affect specific salience network regions in patients with epilepsy with generalized tonic-clonic seizures alone (EGTCSa).

The effects of some antiepileptic drugs on cognition have been observed in previous studies, but those results are heterogeneous, especially given the lack of standardized evaluations on large study samples.

Several studies have previously examined how ASMs may influence cognitive processes in different domains of the brain of patients with epilepsy (attention, memory, psychomotor speed), especially older ASMs. In some studies, older ASMs like phenytoin, carbamazepine, and valproate were reported to have effects on cognition, while in other studies, little change was found. In a review that compared ASMs’ effects on cognitive function in patients with epilepsy, it was noted that most studies used cognitive test batteries and quality of life regarding cognitive domains. Compared with traditional ASMs (carbamazepine, phenytoin, valproic acid), tests using quality-of-life assessments or subjective reports showed no cognitive adverse effects or positive psychotropic effects with newer ASMs (lamotrigine) [18].

By contrast, the present study examined the activity of particular regions of interest in the salience network among patient groups treated with older ASMs, newer ASMs, and without treatment. Researchers have long been concerned about the effect of antiepileptic drugs on cognition, and this approach can provide a new perspective on this issue. In our study, we tried to find out the differences between the old and new generation AEDs by studying the activity of some regions of the salience network of EGTCSa patients.

## 2. Materials and Methods

### 2.1. Participants

The present study included 40 consecutive right-handed patients diagnosed with EGTCSa alone who underwent an EEG in our unit (Elytis Hospital Hope, Iasi, Romania) in the last 3 years and 40 age-matched healthy subjects. In this retrospective case-control study, EEG and clinical data were retrospectively analyzed with the following inclusion and exclusion criteria. We collected EEG data in EDF format for each patient and the selection criteria are described above. Analysis of clinical data was performed using EEG basic reports stored in the EEG device. We included both males and females with an EGTCSa diagnosis, with or without medication. Patients who had a positive history of hemorrhagic or ischemic stroke/tumor/trauma, patients who yielded an abnormal MRI, and patients with EEG recordings for <30 min were all excluded. Structural and focal epilepsies (even though they can coexist with IGEs in rare cases) were excluded from the study. In order to maximize the first EEG epoch selection, smaller recordings were excluded. Specifically, 14 females and 26 males between the ages of 18 and 69 years were recruited. In 14 patients the age of seizure onset could not be found, whereas in 8 patients the onset occurred in early childhood, and in 18 patients the onset commenced after 18 years old. This study was approved by the University of Medicine and Pharmacy ‘Grigore T.Popa’ Iasi ethical committee (approval no. 381/17 January 2024) and it was in accordance with the tenets of the Helsinki Declaration.

### 2.2. EEG Recording

The EEG was performed in a quiet room, using a 19-scalp electrode cap (Cadwell Industries, Inc., Kennewick, WA, USA). The recordings were performed with a sampling rate frequency of 256 Hz and the artifacts were corrected by the technician. The impedances were kept below 5 kΩ and the paper speed was 30 mm/s.

A certified EEG neurologist selected the EEG data according to the following criteria: (i) The presence of the α posterior rhythm; (ii) the absence of sleep stages or drowsiness; (iii) the absence of epileptic discharges; (iv) ≥10 s distance from specific activation maneuvers, including hyperventilation and photic stimulation; and (v) the absence of common artifacts (or as few as possible). The exclusion criteria were as follows: (i) The presence of multiple artifacts (sweat/blinking/electrode/muscle artifacts); (ii) the presence of focal slowing; (iii) the presence of ictal/interictal discharges; (iv) the presence of predominant β rhythm in anterior derivations; and (v) the presence of non-rapid eye movement sleep EEG hallmarks (slow waves, sleep spindles and K-complexes).

### 2.3. EEG Preprocessing

The selected data was imported into the EEGLAB toolbox in v2020MATLAB R2023b (MathWorks, Inc., Natick, MA, USA). The following steps were performed regarding signal preprocessing: (i) Artifacts were manually removed, where 0.5 and 40 Hz were selected for high- and low-pass filtering, respectively; (ii) signals were re-referenced to an average reference; (iii) 2-min segments were selected for each patient (60 epochs, 2 s each) for independent component analysis; (iv) artifactual data was removed (maximum 3 independent components out of 19 for each patient); (v) remaining data of brain activity for every independent component was verified using the activity power spectrum; and (vi) the data was exported into LORETA using a specific plugin (loreta2.0).

In the selected epochs, artifacts such as muscle, eye, sweat, and EKG were visually inspected and removed from the data. Channels with noise were interpolated. Lastly, we classified the signal components in the brain and extra-brain signals based on independent component analysis.

### 2.4. Low Resolution Electromagnetic Tomography (LORETA)

In LORETA, data collected from EEG or MEG (Magnetoencephalography) measurements are used to determine a 3D distribution of electric activity with the optimal similarity in terms of orientation and strength, between neighboring neuronal populations. The inverse solution has zero localization error, which is a method that has been validated by several studies (20 papers from 2001 to 2002) [19].

LORETA offers the advantage of extracting different network components from EEG data, as well as estimating electric neuronal activity in the cortex.

Regarding the head model, LORETA utilizes the Montreal Neurological Institute (MNI coordinates), and the intracerebral volume is represented by 6239 voxels at 5 mm [20,21]. In addition, in LORETA, the following regions of interest from the salience network were defined for both hemispheres: (i) BA 32 (anterior cingulate); (ii) BA 24 (anterior cingulate); and (iii) BA 13 (sublobar insula). In LORETA we defined the number of regions of interest and for each region selected we had the following features: MNI coordinates, lobe, structure, and Brodmann area. Every voxel belonging to a specific region can be selected in this step. The regions of interest selected from the salience network contain MNI coordinates and all the voxels from the same region of interest were averaged in the transformation matrix. The regions of interest defined for both hemispheres are defined in Table 1.

For the present study, patients with epilepsy were divided into four groups: (i) Group 1 (G1), who underwent valproate treatment; (ii) Group 2 (G2), who underwent carbamazepine treatment; (iii) Group 3 (G3), who underwent levetiracetam treatment; and (iv) Group 4, who did not receive treatment. In addition, the control group was also divided into four groups according to age. For each group, a matrix that represented the average activation for each region of interest was obtained for each timeframe in the epoch.

### 2.5. Statistical Analysis

The baseline data statistics were performed in Excel—version 2403 (Microsoft Corporation, Redmond, WA, USA) and the results were expressed in percentages. The χ^2^ test was performed to compare the group with EGTCSa and the control group for both male and female patients.

A Wilcoxon rank-sum test (the Mann-Whitney U test) was performed to assess the equality of medians. This test is a non-parametric version of the *t*-test for independent samples. The *t*-test for independent samples could not be applied here, since the sample values were not distributed normally and the sample volume was relatively low. By using the Kolmogorov normality test, we observed that the datasets were not normally distributed at a significance level of 0.05. In total, three statistical tests were considered here in this study. For all three tests, the null hypothesis is the epilepsy group and the control group has equal medians. The alternative hypotheses were as follows: (i) the two groups have different medians according to a two-tailed test); (ii) the median for the epilepsy group is lower compared with that of the corresponding median of the control group (left tails); and (iii) the epilepsy group has a larger median compared with that in the control group (right tails). The maximal *p*-value threshold considered in the present study is α = 0.05. The Wilcoxon rank-sum test was applied to all 6 features of interest (regions of interest) in every group with EGTCSa defined (G1–G4) and controls. We compared median values in G1–G4 of the 6 features with median values in the corresponding control groups.

## 3. Results

**Figure 1:**
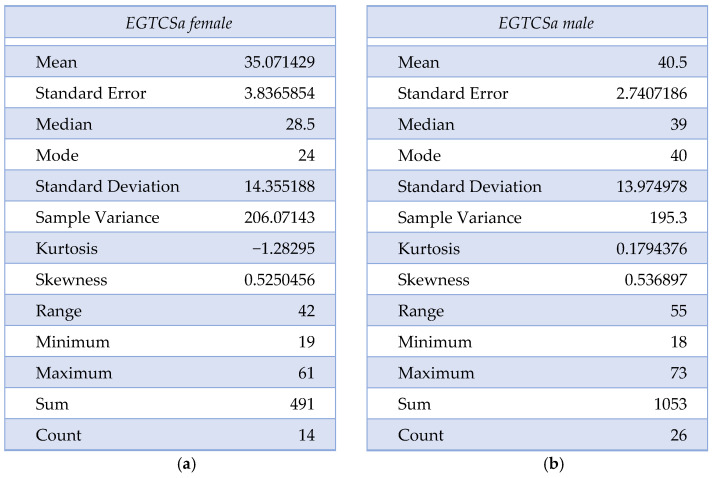
Descriptive statistics for epilepsy group (EGTCSa): (**a**) Description of EGTCS group, female patients; (**b**) Description of EGTCS group, male patients.

**Figure 2:**
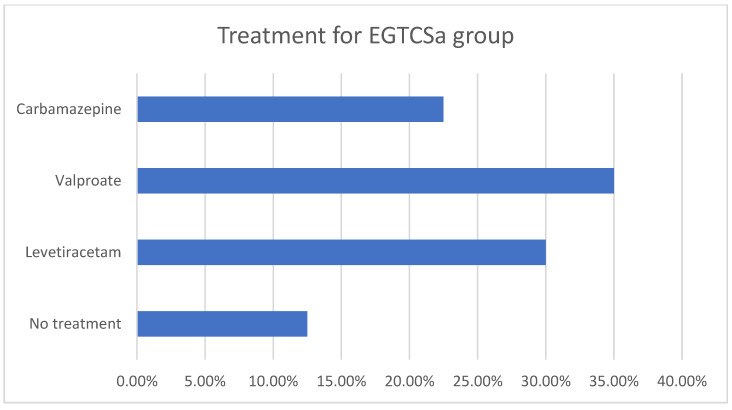
Descriptive statistics for epilepsy group (EGTCSa). Antiseizure medication in the epilepsy group: 12.5% were without treatment, 30% with levetiracetam, 35% with valproate and 22.5% with carbamazepine.

A statistical difference (the maximal *p*-value threshold considered here is α = 0.05) could not be found between the two groups regarding age and sex distribution, obtaining *p* = 0.07 according to the χ^2^ test. The mean age for the epilepsy group was 35.07 ± 14.16 for female patients and 40.5 ± 13.97 for male patients. In the control group, the mean age was 36.27 ± 9.63 for female patients and 41.44 ± 18.74 for male patients. The descriptive analysis for the epilepsy group is summarized in Figure 1a,b and Figure 2. Although the skewness is similar between males and females with EGTCSa, there is a difference in kurtosis between the two groups and it indicates a difference in distribution at central values (Figure 1). In Figure 2 the EGTCSa treatment in this group is detailed. In addition to the previous description, a percentage of 7.5% of the total is male without medication and 5% is female without medication. The small sample of this group (without treatment) could be a source of bias.

The clinical data for patients with EGTCSa is summarized in Table 2.

In G1 and G2, significantly different activity was found in the regions defined compared with those in the control group. In G1, patients treated with valproate had lower values compared with those in the control group for all regions defined in the anterior cingulate cortex and right sub-lobar insula. In G2, the patients treated with carbamazepine had lower values for every region defined (anterior cingulate cortex and sub-lobar insula) compared to the control group. Compared with those in the control group, patients treated with levetiracetam had higher values for the right sub-lobar insula, whilst the group without treatment had no significant statistical differences.

The statistically significant *p*-values found for every group are summarized in Table 3 (red).

## 4. Discussions

In this study, differences in the regions of the salience network were detected in patients with active epilepsy treated with ASMs and in healthy control individuals. A possible explanation is that the ASM can influence cognitive processes, which may guide the selection processes of the optimal ASM in the future.

An understanding of the neural mechanisms underlying the administration of ASMs may be brought about by investigating the nature of alterations in the connectivity parameters. Marked differences were found in this study in regions from the salience network in patients with EGTCSa treated with valproic acid and carbamazepine, whereas little difference could be found compared with control individuals in patients treated with levetiracetam, and no differences could be found in the group without treatment compared with control individuals. It could be speculated that older ASMs may influence key regions in the salience network more than recent ASMs and therefore the cognition of such patients receiving older ASMs. However, larger studies are necessary to confirm this hypothesis, where further information regarding the interactions between the salience network and other resting state networks will likely provide novel insights in this area.

From a network perspective, these approaches and results may assist clinicians in selecting the appropriate ASM for their patients. The differences between the newer and older ASMs obtained need to be further evaluated with larger cohorts since the study has a small sample size. Especially for drugs like carbamazepine and valproate, the effects on different networks have to be evaluated, as well as cognitive function in different domains, since these drugs are prescribed for certain epilepsy conditions as first-line therapies.

The effects of psychotropic medications on EEG have been previously studied using numerical parameters derived from EEG signals, such as the ratio of the power spectrum at each frequency. As a result of these EEG findings, clinicians were able to select the appropriate medication based on patient comorbidities [22].

Conventional ASMs, including phenytoin, carbamazepine, and phenobarbital, are associated with slower posterior rhythms in patient groups compared with those receiving other ASMs or groups not treated with ASMs. Interactions between ASMs and epileptic networks upstream of their effects on brain activity may serve to be one underlying mechanism [23].

Studying into cognitive side-effects of ASM is a relatively novel research area of interest. It has been previously demonstrated that cognitive side-effects form one of the most important problems associated with chronic ASM use [24].

In resting-state fMRI, the insula and anterior cingulate cortex were found to anchor the salience network. These regions are important for interoceptive awareness. Therefore, insular functional connectivity in the salience network at rest is associated with individual variations in interoceptive awareness [25].

Research into resting networks and their activity following medication represents a recent area of research that can provide clinicians with novel information for future research directions. In generalized epilepsy syndromes, several resting state networks were analyzed such as default mode network and salience network.

With EEG-fMRI, a previous study was performed on 12 patients with genetic generalized epilepsy and generalized spike-wave discharges (GSWDs) with the aim of determining which networks are involved in the generation and evolution of generalized spike-wave discharges [25]. Deactivations in the default mode network (DMN) and dorsal attention network (DAN), activations in the salience network (SN), and thalamus were found to precede the onset of discharges on EEG by several seconds. However, heterogeneity in the study’s cohort was observed because different generalized epilepsy syndromes were included [26].

Studies describing differences in the default mode network in patients with generalized epilepsy, specifically in the middle frontal gyrus also exist, which may lead to a novel approach for treating generalized epilepsy syndromes. Deepening the understanding of the pathophysiology and connectivity of particular regions may improve such treatment approaches [27].

Right anterior insula-DMN coupling increases with cognitive control, a dynamic interaction process that can be impaired by damage to the salience network. When attention is externally focused, the right anterior insula signals the attentional capture of the salient stimuli and interacts with the DMN to reduce its activity [28].

In a previous double-blind, placebo-controlled study, the effects of ASM discontinuation on cognitive functions were assessed. The majority of patients were treated with carbamazepine and valproate. The results suggested that drug discontinuation improved complex cognitive processing under time pressure [29]. Another previous comparison of the effects between carbamazepine (CBZ) and lamotrigine (LTG) found that carbamazepine has a worse cognitive profile compared to lamotrigine. The study included patients receiving monotherapy with LTG and CBZ and those who were treated with LTG performed better on phonemic fluency tests [30].

The effects of discontinuing phenytoin, carbamazepine, and valproate were previously examined in a double-blind, prospective, placebo-controlled study in 58 patients with active epilepsy. The results found an improvement in simple motor skills after discontinuation [31]. Furthermore, we demonstrated the effect of older ASMs like carbamazepine and valproic on the salience network, supporting previous studies.

As an alternative, levetiracetam may improve cognitive functions. An increase in vigilance, psychomotor speed, concentration, and remote memory was previously reported. A number of factors led to the negative ratings, including lack of self-control, restlessness, sleep problems, and particularly aggressive behavior [32]. According to our study, patients treated with levetiracetam had little influence on salience networks, with higher values for the sublobar insula, supporting the idea that this ASM can improve cognitive abilities. In Table 4 the ASMs effects reported in previous studies and related to our findings are summarized.

There is, however, the potential for all ASMs to exert negative effects on cognitive function. To achieve therapeutic success, it is of vital importance to understand the negative cognitive effects of a variety of ASMs [33].

ASMs can exert a global effect on the physiology of the brain, which is most likely reflected in functional brain activity. In a previous study on 10 patients with focal epilepsy, EEG-fMRI showed that functional connectivity between resting state networks increased after ASM withdrawal [34].

For drug-resistant epilepsy, a previous study has proposed the use of network biomarkers for predicting cognitive deficits, postsurgical outcomes, and localized surgical targets. As one example, temporolimbic and thalamic structural connectivity were evaluated to predict the recurrence of seizures following surgery. However, it remains unclear how neuroimaging biomarkers can be translated to the bedside of patients due to a lack of clinical and prospective studies [35].

As a result of our findings, we are opening up new perspectives on this old topic, and we might be able to gain better insights into the long-term effects of ASMs through network studies. The knowledge in this area could be improved by multicentric studies.

The results of a larger study can contribute to a better understanding of this field, which can have an unequivocal impact on clinical practice. For patients with epilepsy, monitoring cognitive side effects of ASMs has a significant impact on quality of life, and should be carefully indicated to those with long-term recommendations. There may be some benefit in incorporating several cognitive domains to follow in treated patients, eventually becoming a guideline recommendation for specific tests in specific ASMs.

A limitation of this study is the small number of patients requiring non-parametric statistical analyses, especially after subdividing by medication, and the high bias risk. The presence of a small number of patients without medication is another potential bias. A limitation of the systematic review is its need to include more detailed literature on ASM’s effects on particular cognitive domains. Recruiting patients from multiple epilepsy centers is imperative for improved reliability and the possibility of including other generalized epilepsy syndromes. High-quality and rigorous studies may provide definitive proof of the cognitive effects of ASMs. As a result of our findings regarding ASM’s effects on the salience network, clinicians as well as researchers may find our findings useful. Neuromodulation in certain regions of interest in selected patients may help improve certain dysfunctions and represent a potential future direction.

## 5. Conclusions

According to the present findings, patients with epilepsy treated with older ASMs, such as valproate and carbamazepine, had lower values compared with controls in salience network regions (anterior cingulate cortex and sublobar insula). By contrast, patients treated with newer ASMs, such as levetiracetam, had higher sub-lobar insula values. There was no difference between the group without treatment and the control group.

In light of these findings, they may be of importance for clinically choosing the most appropriate ASMs, since they suggest that ASMs can influence neural processes that lead to cognitive functions. As a result of this approach, novel lines of research can be developed, especially in patients with epilepsy who are resistant to drugs or those requiring the discontinuation of ASM treatment. There may be potential for evaluating the connectivity of resting-state networks in patients who have drug-resistant epilepsy. As a future approach, resting state network analysis may be a superior method for analyzing the effects of ASM withdrawal.

As a conclusion, studies have shown that motor performance improves after stopping certain older anticonvulsants, and newer antiepileptic drugs have a better cognitive profile than older ones. It is novel to approach this subject from the perspective of resting neural networks, such as the salience network, which shows consistent changes concerning the old generation of anticonvulsant medications.

Several limitations must be emphasized for this study. A small number of patients limited to non-parametrical statistical tests, especially after division according to antiseizure medication, was used. In addition, the difficulty in dividing the groups according to the number of ASMs was another limitation, since there were few patients receiving dual and triple ASMs in this study. The small number of electrodes and the absence of inferior temporal line electrodes also represent another limitation for electrical source imaging performed in LORETA. Larger studies are necessary to compare interactions between resting state networks, such as DMN, DAN, and salience networks, and the effects of ASMs on such interactions.

## Figures and Tables

**Table 1:** Regions of interest selected from the salience network.

Lobe	Structure	Brodmann Area	X-MNI	Y-MNI	Z-MNI
Limbic lobe	Anterior cingulate	32	−10	35	10
Limbic lobe	Anterior cingulate	32	−5	20	−10
Limbic lobe	Anterior cingulate	32	10	35	−10
Limbic lobe	Anterior cingulate	32	5	20	−10
Limbic lobe	Anterior cingulate	24	−5	25	−5
Limbic lobe	Anterior cingulate	24	−5	30	−5
Limbic lobe	Anterior cingulate	24	5	25	−5
Limbic lobe	Anterior cingulate	24	5	30	−5
Insula	Sublobar	13	−40	−20	−10
Insula	Sublobar	13	−45	10	−5
Insula	Sublobar	13	40	−25	−5
Insula	Sublobar	13	45 ^1^	10 ^1^	−5 ^1^

^1^ MNI (Montreal Neurological Institute) coordinates.

**Table 2:** The demographics and clinical characteristics of patients with EGTCSa.

Age * (years)	38.8 ± 14.16
Gender	Male: 65%
	Female: 35%
Duration of epilepsy	<5 years: 22.5%
	>5 years: 60%
	Unknown: 17.5%
Age at seizure onset (in years)	<18: 27.5%
	18–40: 25%
	>40: 20%
	Unknown: 27.5%
Febrile convulsions history	(+) 10%
	(−) 55%
	Unknown: 35%

* At the EEG recording.

**Table 3:** *p*-values obtained for all four groups (in red significant statistical difference *p* < 0.05).

Groups	Region of Interests							
Group 1 (Valproate)		feature	1	2	3	4	5	6
	(H1)	*p*-value	0.0106	0.0008	0.0147	0.0034	0.9340	0.0387
	(H1left)	*p*-value	0.0053	0.0004	0.0074	0.0017	0.5333	0.0193
Group 2 (Carbamazepine)		feature	1	2	3	4	5	6
	(H1)	*p*-value	0.0009	0.0031	0.0065	0.0112	0.0838	0.0330
	(H1left)	*p*-value	0.0004	0.0016	0.0033	0.0056	0.0419	0.0165
Group 3 (Levetiracetam)		feature	1	2	3	4	5	6
	(H1)	*p*-value	0.5229	0.1351	0.5403	0.1934	0.8072	0.0533
	(H1right)	*p*-value	0.2614	0.0676	0.2702	0.0967	0.5967	0.0266
Group 4 (No treatment)								
	Without significant statistical differences							

Significance level 0.05.

**Table 4:** Effects of ASMs studied in previous studies.

Medication	Effect on Various Domains
Carbamazepine Valproate	Complex cognitive processing under time pressure improvement after discontinuation [29]
Carbamazepine Lamotrigine	Patients treated with lamotrigine performed better on phonemic fluency tests Carbamazepine has a worse cognitive profile [30]
Phenytoin Carbamazepine Valproate	An improvement in simple motor skills after discontinuation [31]
Levetiracetam	An increase in vigilance, psychomotor speed, concentration and remote memory [32]

## Data Availability

The original contributions presented in the study are included in the article, further inquiries can be directed to the corresponding author.

## Abbreviations

ASM	antiseizure medication
BA	Brodmann area
CBZ	carbamazepine
DAN	dorsal attention network
DMN	default mode network
EEG	electroencephalogram
EGTCSa	epilepsy with generalized tonic-clonic seizures alone
GE	generalized epilepsy
GSWDs	generalized spike-wave discharges
IGE	idiopathic generalized epilepsies
SN	salience network
